# The prevalence of pre-diabetes and diabetes and their associated factors in Northeast China: a cross-sectional study

**DOI:** 10.1038/s41598-019-39221-2

**Published:** 2019-02-21

**Authors:** Rui Wang, Peng Zhang, Zhijun Li, Xin Lv, Hongfei Cai, Chunshi Gao, Yuanyuan Song, Yaqin Yu, Bo Li, Youbin Cui

**Affiliations:** 1 Department of Thoracic Surgery, The First Hospital of Jilin University, Changchun, 130021 China; 2 Department of Neurology, Stroke Center, the First Hospital of Jilin University, Chang Chun, 130021 China; 3 Department of Epidemiology and Biostatistics, Jilin University School of Public Health, 1163 Xinmin Street, Changchun, Jilin 130021 China; 4 Department of Epidemiology and Biostatistics, Beihua University School of Public Health, Chang Chun, China

## Abstract

This study investigates the prevalence of pre-diabetes and diabetes and their associated risk factors among adults in Northeast China. A multistage stratified cluster sampling method was used to select adults from Jilin Province. Out of an initial recruitment of 23,050 individuals, 21,435 participants completed an interview and medical examination. The estimated prevalence of diabetes and pre-diabetes were 9.1% and 19.8%, respectively. The prevalence of hypertension, dyslipidemia, and obesity were the highest in participants with previously diagnosed diabetes. Participants who were previously diagnosed with diabetes were more likely to be aware of their hypertension and dyslipidemia status. Participants who were older, male, more educated, or who were widows or widowers were at greater risk for pre-diabetes. Similarly, those who were current drinkers or smokers, had higher BMI or waist circumference, had a family history of diabetes, or who reported they lived in urban areas or had low physical activity levels had increased pre-diabetes risk. The observed levels of diabetes and pre-diabetes in this study indicate that the medical authority needs to focus more attention in this area, and that health monitoring is essential to improving the health awareness of its residents.

## Introduction

Diabetes mellitus is one of the leading causes of death and disability worldwide^1,2^. In 2014, the International Diabetes Federation estimated that the number of people with diabetes will rise from 387 million to 592 million by 2035^3^. Diabetic patients have a substantially elevated risk of cardiovascular disease and likewise cardio metabolic syndrome is associated with an increased risk of diabetes^4^. Diabetes has also been linked to hypertension and hyperlipidemia, with the pharmacological treatment of hypertension in diabetic patients significantly reducing macro vascular complications^5,6^, and highlighting the importance of managing blood pressure and blood lipids in the diabetic population.

The prevalence of diabetes has reached over 30% in some countries, most notably in the western pacific islands, such as Tokelau (37.5%), Federated States of Micronesia (35%) and the Marshall Islands (34.9%)^2^, although In 2013 the overall prevalence of diabetes in mainland China was 10.9%^7^, The prevalence of pre-diabetes in China is 35.7%^8^, much higher than in some other countries such as Saudi Arabia (6.8%), India (6.3%), and the southern cone of Latin America (17.8%)^3,9,10^. It is not clear whether pre-diabetes increases the risk of hypertension and hyperlipidemia. To provide reliable data and suggestions for prevention, this study aims (1) to estimate the prevalence of diabetes and pre-diabetes, and their influencing factors, in the adult population in the Jilin province; and (2) to describe the association between diabetes and other chronic diseases in the same population.

## Method

### Study design and population

This is the first large face-to-face cross-sectional interview based study in the Jilin Province, Northeast China. This study adopted a multistage stratified cluster sampling method to select adult permanent residents in the province: Firstly, nine administrative regions were selected covering the whole province (Changchun, Jilin City, Siping, Liaoyuan, Tonghua, Baishan, Songyuan, Baicheng and Yanbian), which all have a large responsibility for health care. Secondly, from each of the nine regions, clusters of four districts, or counties, were randomly selected based on probability proportional to size (PPS) sampling. According to the National Bureau of Statistics of China, each selected district or county in this survey is divided into urban and rural areas^11^. Thereafter, four or five communities were randomly taken out from the urban and rural strata by PPS. Finally, one adult was randomly chosen from each household of the communities mentioned above (pregnant individuals were not included). In total, 23,050 adult subjects were recruited from 32 districts or counties, 95 towns or communities, and 45 units in the Jilin Province^11^. In total 21,435 participants completed the interview (response rate: 92.2%). After excluding invalid questionnaires and abnormal data (for example an indicated weight of 12 kg), 18,362 participants were included in the study. The study was approved by the Ethics Committee of Jilin University School of Public Health, and written informed consents were obtained from all the subjects in the survey. We confirm that all methods were performed in accordance with relevant guidelines and regulations.

### Questionnaire investigation

116 trained investigators conducted the interview in local health centers and communities using structured questionnaires. The questionnaires covered subjects’ socio-demographic characteristics and health related information. Each questionnaire was examined by the interviewer through a parallel double entry system after being completed by participants^12^.

### Physical examination

A physical examination was conducted by trained investigators, and consisted of anthropometric measurements including height, weight, blood pressure, and fasting blood glucose (FBG & OGTT) and blood lipid levels. Blood samples were collected from participants in the morning after an overnight fast of 10 h or more. The BaiAnkang fingertip blood glucose monitor machine (Bayer, Beijing, China) was used to measure FBG levels by collecting a small drop of blood from a finger of the participant onto a strip of paper. Post-fast blood samples were also drawn by venipuncture to measure blood lipid concentration. After collection, the samples were placed in a cold chain system before being collectively transported to a central laboratory at Jilin University.

### Definitions of major Variables

Diabetes: FBG ≥7.0 mmol/L, oral glucose tolerance test (OGTT-2 h) ≥11.0 mmol/L plasma glucose or self-reported use of anti-diabetic medication during the 2 weeks prior to the examination^13^.

Pre-diabetes: FBG from 6.1 to 6.9 mmol/l, OGTT-2 h: 7.8–10.9 mmol/l^14^.

Hypertension: systolic pressure >140 mmHg or diastolic pressure >90 mmHg^15^.

Dyslipidemia: Total cholesterol (TC) >5.18 mmol/L and/or (TG) triglyceride TG > 1.70 mmol/L and/or high-density lipoprotein cholesterol (HDL-C) <1.04 mmol/L and/or low density lipoprotein cholesterol (LDL-C) >3.37 mmol/L^12^. (Having a history of dyslipidemia and Hypertension disease in the past one year, and/or currently receiving treatment with lipid-lowering medications was regarded as dyslipidemia and Hypertension in this study).

Body Mass Index (BMI): For the Chinese population, obesity is defined as a BMI of ≥28 kg/m^2^, and overweight as a BMI of 24–27.9 kg/m^2 ^^16^.

Central obesity: waist circumference (WC) >80 cm for females and WC >85 cm for males^17^.

Age groups: according to the criteria of age classification by WHO reported in 2012, age range is divided into three groups: young (18–44 years), middle (44–59 years) and old (≥60 years)^16^.

Other factors: A smoker was defined as a person who smoked at least one cigarette per day within the last 30 days, and drinker was a person who consumed more than one alcoholic drink per week. Participants were divided into those that “sometimes exercise”, with an exercise frequency of one or two times a week; those who exercised more than three times a week were defined as “exercise frequently”; while those who didn’t or seldom exercised were defined as those that “never or rarely exercise”^18^.

### Statistical analyses

Post stratification adjustment was used to make the sample representative of the provincial population^11^. The adjustment was made according to the distribution of gender and age groups in the census of the adult population of Jilin Province in 2010. All the data were input into Epidata 3.0, and analyzed with SPSS (ver. 22.0; IBM Corp, Armonk, NY, USA). Continuous variables were expressed by mean and standard deviation; and categorical variables were presented as frequencies. Odds ratios (ORs) and 95% confidence intervals (CIs) were calculated by a logistic regression model. *P* < 0.05 was considered to be statistically significant.

## Results

Of the 23,050 participants initially recruited, 18,362 participants aged from 18 to 79 were included in this study. The estimated prevalence of diabetes and pre-diabetes were 9.1% and 19.8% respectively. Among the diabetic participants, 68.9% were previously diagnosed diabetic patients and 31.1% were newly diagnosed in this study. Table 1 shows the socio-demographic characteristics of the study population according to glycemic status.

**Table 1:** Characteristics of Northeastern Chinese adults by glycemic status.

Characteristics		Glycemic status				Χ^2^/F*	p
		Normal glucose regulation (n, %)	Pre-diabetes (n, %)	Previously-diagnosed diabetes (n, %)	Newly-diagnosed diabetes (n, %)		
Sex	Men	5474 (42.9)	2083 (57.2)	739 (54.9)	364 (59.8)	279.13	<0.001
	Women	7291 (57.1)	1558 (42.8)	608 (45.1)	245 (40.2)		
Region	Rural	6337 (49.6)	1485 (40.8)	675 (50.1)	287 (47.1)	92.16	<0.001
	Urban	6428 (50.4)	2156 (59.2)	672 (49.9)	322 (52.9)		
Age	Young	5880 (46.1)	1273 (35.0)	130 (9.7)	137 (22.5)	972.40	<0.001
	Middle	4702 (36.8)	1600 (43.9)	652 (48.4)	307 (50.4)		
	Old	2183 (17.1)	768 (21.1)	565 (41.9)	165 (27.1)		
BMI	Normal	6450 (50.5)	1291 (35.5)	415 (30.8)	188 (30.9)	692.68	<0.001
	Underweight	665 (5.2)	86 (2.4)	19 (1.4)	11 (1.8)		
	Overweight	4083 (32.0)	1531 (42.0)	584 (43.4)	273 (44.8)		
	Obesity	1567 (12.3)	733 (20.1)	329 (24.4)	137 (22.5)		
Central obesity	No	7436 (58.6)	1487 (41.1)	313 (23.8)	180 (30.2)	919.80	<0.001
	Yes	5260 (41.4)	2130 (58.9)	998 (76.1)	417 (69.8)		
Education	Primary school and below	3728 (29.2)	906 (24.9)	563 (41.8)	210 (34.5)	204.67	<0.001
	Junior middle school	3695 (28.9)	1000 (27.5)	350 (26.0)	170 (27.9)		
	Senior middle school	3203 (25.1)	1130 (31.0)	314 (23.3)	159 (26.1)		
	Under graduate and above	2139 (16.8)	605 (16.6)	120 (8.9)	70 (11.5)		
Occupation	Intelligence	2632 (20.6)	730 (20.0)	169 (12.5)	105 (17.2)	480.17	<0.001
	Manual	7318 (57.3)	2020 (55.5)	594 (44.1)	331 (54.4)		
	Retired	1195 (9.4)	504 (13.8)	376 (27.9)	82 (13.5)		
	Others	1620 (12.7)	387 (10.6)	208 (15.4)	91 (14.9)		
Marriage	Married/cohabitation	10837 (85.2)	3170 (87.1)	1205 (89.5)	543 (89.2)	164.07	<0.001
	Single	1055 (8.3)	211 (5.8)	11 (0.8)	14 (2.3)		
	Divorced/Separated	238 (1.9)	71 (2.0)	23 (1.7)	18 (3.0)		
	windowed	599 (4.7)	189 (5.2)	108 (8.0)	34 (5.6)		
Drink	No	8994 (70.5)	2209 (60.7)	1045 (77.6)	358 (58.8)	204.26	<0.001
	Yes	3771 (29.5)	1432 (39.3)	302 (22.4)	251 (41.2)		
Smoking	Never	8178 (64.1)	2005 (55.1)	826 (61.3)	313 (51.4)	218.58	<0.001
	Now	3719 (29.1)	1288 (35.4)	328 (24.4)	233 (28.3)		
	Once	868 (6.8)	348 (9.6)	193 (14.3)	63 (10.3)		
Exercise	Never or rare	6198 (48.6)	1543 (42.4)	402 (29.8)	267 (43.8)	470.22	<0.001
	Sometimes	3180 (24.9)	904 (24.8)	219 (16.3)	125 (20.5)		
	Frequently	3387 (26.5)	1194 (32.8)	726 (53.9)	217 (35.6)		
Family history of diabetes	No	11170 (87.5)	3120 (85.7)	904 (67.1)	494 (81.1)	416.75	<0.001
	Yes	1595 (12.5)	521 (14.3)	443 (32.9)	115 (18.9)		
Hypertension	No	8776 (68.8)	2020 (55.5)	507 (37.6)	270 (44.3)	732.33	<0.001
	Yes	3989 (31.2)	1621 (44.5)	840(62.4)	339 (55.7)		
Dyslipidemia	No	8806 (39.0)	1928(53.0)	578 (42.9)	217 (35.6)	780.60	<0.001
	Yes	3959 (31.0)	1713 (47.0)	769 (57.1)	392 (64.4)		
Blood sugar (mmol/l)	Fasting plasma glucose	4.65 ± 0.58	6.00 ± 0.36	8.00 ± 3.21	8.64 ± 2.09	6316.98	<0.001
	OGTT-2 h plasma glucose	5.56 ± 0.98	8.80 ± 0.86	10.82 ± 4.98	15.41 ± 5.66	370.78	<0.001
Blood lipid	TG	1.70 ± 1.38	2.37 ± 2.14	2.79 ± 2.53	3.42 ± 3.34	71.31	<0.001
(n = 11811)	TC	4.79 ± 1.03	5.09 ± 1.08	5.31 ± 1.31	5.40 ± 1.31	23.07	<0.001
(mmol/l)	LDL-C	2.88 ± 0.86	3.06 ± 0.92	3.18 ± 1.01	3.09 ± 1.01	22.17	<0.001
	HDL-C	1.43 ± 0.39	1.32 ± 0.37	1.23 ± 0.32	1.25 ± 0.36	20.45	<0.001
Blood pressure	SBP	129 ± 21	135 ± 21	141 ± 23	140 ± 23	11.75	<0.001
(mmHg)	DBP	79 ± 11	82 ± 12	82 ± 12	85 ± 13	7.16	<0.001

*Qualitative data was used Χ^2^test and Quantitative data used F test.

The newly-diagnosed group was younger, with a larger proportion of men, manual laborers, singles or widows/widowers, current drinkers or smokers, a family history of diabetes and a low frequency of physical exercise compared to the participants with previously diagnosed diabetes. In addition, the newly diagnosed participants had a higher WC, blood sugar levels (both FBG and OGTT-2 h plasma glucose), TG and DBP levels than those of the previously diagnosed group.

**Figure 1:**
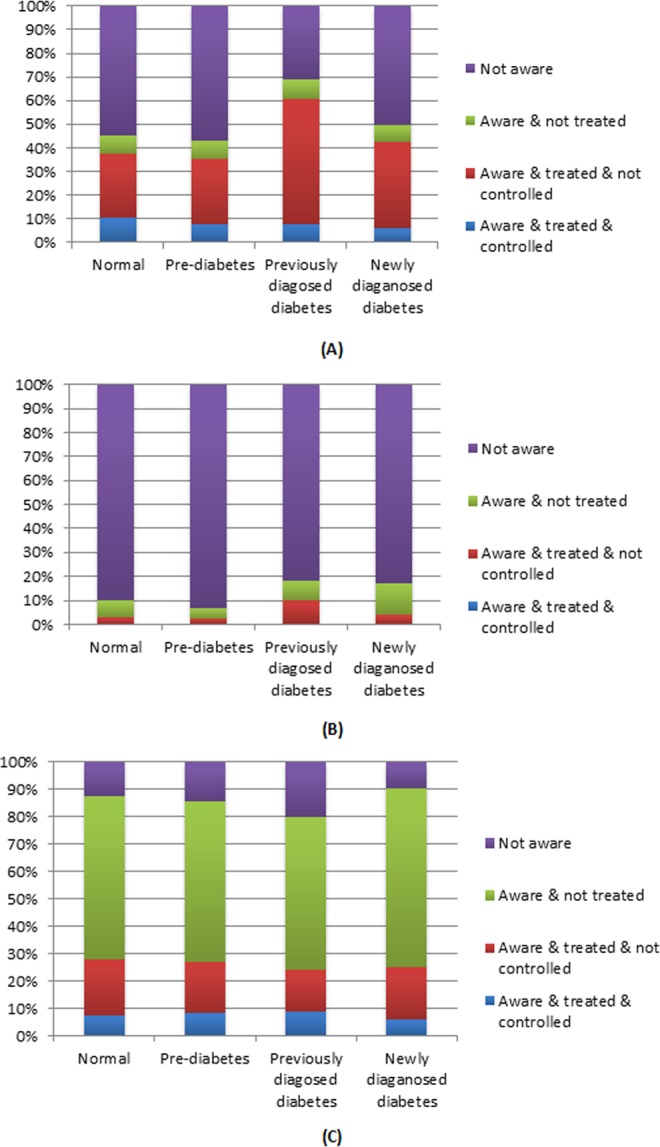
Awareness, treatment and control of hypertension (**A**), dyslipidemia (**B**) and obesity (**C**) by glycemic status.

The prevalence of hypertension, dyslipidemia, and obesity were highest in participants with previously-diagnosed diabetes, but participants tended to be more aware of their hypertension and dyslipidemia status compared to other groups. As shown in Table 2, among participants who were aware of their respective disease condition, the previously-diagnosed diabetes group was most likely to be receiving treatment, but overall the proportion of participants receiving measures to control their blood pressure, lipid levels and obesity was not high. Figure 1 shows the status of awareness, treatment and control, in hypertension, dyslipidemia and obesity. It is worth noting that the prevalence of dyslipidemia was at a particularly high level, but the rates of awareness, treatment and control were low.

**Table 2:** Awareness, treatment and control of hypertension, dyslipidemia and obesity by glycemic status.

Items*		Glycemic status			
		Normal glucose regulation (n, %) (n = 12765)	Pre-diabetes (n,%) (n = 3641)	Previously-diagnosed diabetes (n, %) (n = 1347)	Newly-diagnosed diabetes (n %) (n = 609)
Hypertension	Prevalence	3989 (31.2)	1621 (44.5)	840 (62.4)	339 (55.7)
	Awareness	1793 (44.9)	693 (42.8)	578 (68.8)	169 (49.9)
	Treatment	1503 (83.8)	569 (82.1)	510 (88.2)	143 (84.6)
	Control	411 (27.3)	127 (22.3)	63 (12.4)	20 (14.0)
Dyslipidemia	Prevalence	3959 (31.0)	1713 (47.0)	769 (57.1)	217 (35.6)
	Awareness	395 (10.0)	119 (6.9)	140 (18.2)	37 (17.1)
	Treatment	121 (30.6)	45 (37.8)	75 (53.6)	9 (24.3)
	Control	6 (5.0)	2 (4.4)	3 (4.0)	0 (0)
Obesity	Prevalence	1567 (12.3)	733 (20.1)	329 (24.4)	137 (22.5)
	Awareness	1368 (87.3)	629 (85.8)	263 (79.9)	124 (90.5)
	Treatment	439 (32.1)	196 (31.2)	79 (30.0)	34 (27.4)
	Control	114 (26.0)	63 (32.1)	29 (36.7)	8 (23.5)

*“Awareness” refers to how many people in the total number of patients know their condition. “Treatment” refers to how many people who know their condition and take treatment measures. “Control” refers to how many of the people who have taken measures have been effectively controlled.

The results of Multivariable logistic regressions among the four groups are shown in Table 3. Older age, male gender, urban residents, widows or widowers, current drinkers or smokers, higher BMI and WC, with an education at undergraduate level and above, having a family history of diabetes and doing physical exercise with low frequency, were all indicators of an increased risk of pre-diabetes. Similarly, for the previously diagnosed diabetes group and the newly diagnosed diabetes group, older age, male gender, higher BMI and WC, not married, being current drinkers, and having a family history of diabetes were risk factors.

**Table 3:** The comparison among different blood glucose groups.

		Pre-diabetes vs. normal			Previously diagnosed diabetes vs. normal			Newly diagnosed diabetes vs. normal		
		OR	95% CI	*P*	OR	95% CI	*P*	OR	95% CI	*P*
Age	Young	1			1			1		
	Middle	1.41	1.38–1.66	<0.001	4.28	3.44–5.24	<0.001	2.35	1.88–2.94	<0.001
	Old	1.68	1.46–1.93	<0.001	5.93	4.64–7.56	<0.001	2.77	2.06–1.62	<0.001
Sex	Men	1			1			1		
	Women	0.65	0.59–0.72	<0.001	0.63	0.53–0.73	<0.001	0.51	0.41–0.64	<0.001
Region	Urban	1			1			1		
	Rural	0.71	0.65–0.77	<0.001	1.12	0.97–1.29	0.115	0.78	0.65–0.95	0.011
BMI	Normal	1			1			1		
	Underweight	0.74	0.58–0.94	<0.001	0.73	0.45–1.18	0.197	0.81	0.44–1.52	0.514
	Overweight	1.52	1.37–1.69	<0.001	1.16	0.98–1.37	0.090	1.48	1.16–1.87	0.001
	Obesity	1.83	1.59–2.09	<0.001	1.53	1.25–1.87	<0.001	1.79	1.34–2.39	<0.001
Education	Primary school and below	1			1			1		
	Junior middle school	1.11	0.96–1.28	0.165	0.97	0.81–1.16	0.751	0.95	0.75–1.20	0.658
	Senior middle school	0.94	0.84–1.05	0.292	0.78	0.60–1.03	0.075	0.79	0.56–1.11	0.176
	Under graduate and above	1.20	1.08–1.34	0.001	1.40	1.19–1.65	<0.001	1.23	0.98–1.55	0.078
Occupation	Intelligence	1			1			1		
	Manual	1.11	0.99–1.25	0.079	0.94	0.75–1.18	0.607	0.95	0.72–1.26	0.733
	Retired	1.14	0.97–1.35	0.109	1.50	1.17–1.92	0.002	0.91	0.63–1.31	0.616
	Others	0.94	0.80–1.11	0.473	1.01	0.77–1.32	0.944	1.23	0.98–1.55	0.606
Marriage	Married/cohabitation	1			1			1		
	Single	1.22	0.89–1.68	0.215	3.22	1.50–6.95	<0.001	3.32	1.60–6.91	0.001
	Divorced/Separated	0.74	0.58–0.94	0.012	2.15	1.11–4.19	<0.001	1.93	0.98–3.79	0.056
	windowed	1.52	1.37–1.69	<0.001	2.68	1.44–4.98	0.002	2.04	1.17–1.87	0.012
Drink	No	1						1		
	Yes	1.18	1.07–1.30	0.001	0.72	0.61–0.85	<0.001	1.28	1.05–1.57	0.017
Smoke	Never	1						1		
	Now	1.15	1.04–1.26	0.007	1.01	0.86–1.18	0.940	1.22	0.99–1.50	0.057
	Once	1.10	0.95–1.28	0.195	1.33	1.08–1.62	0.006	1.01	0.75–1.37	0.944
Exercise	Never or rarely	1			1			1		
	Sometimes	0.98	0.87–1.09	0.650	0.44	0.38–0.51	<0.001	0.93	0.73–1.19	0.572
	Often	0.89	0.81–0.98	0.021	0.66	0.55–0.79	<0.001	0.88	0.72–1.07	0.202
Family history	No	1			1			1		
	Yes	1.15	1.03–1.28	0.016	4.43	3.84–5.10	<0.001	1.85	1.49–2.30	<0.001
Central obesity	No	1			1			1		
	Yes	1.33	1.20–1.47	<0.001	2.61	2.19–3.12	<0.001	2.06	1.62–2.62	<0.001

## Discussion

The prevalence of diabetes in this representative population is 9.1%. According to a previous review, the prevalence of unspecified type diabetes was 10% (95%CI: 9–12%) from 42 different cohorts^19^, which is just slightly higher than the prevalence of diabetes in this study. In recent years, an increasing number of studies have begun to focus on pre-diabetes. An African study reported a prevalence of pre-diabetes at 13.8% in 2016^20^,and a Norwegian study indicated that the age-standardized prevalence of pre-diabetes among men was about 3.3–3.4%, and 2.3–2.7% for women^21^. In this study, the level of pre-diabetes reached 19.8%, over twice the number of participants with diabetes. In a nationally representative sample of 46,239 Chinese adults in 2008, the prevalence of pre-diabetes was 15.5% (16.1% among men and 14.9% among women), which is lower than our observations^22^. Given the seemingly increasing rate of pre-diabetes in China, and that it is a high risk factor for the development of diabetes, the Chinese government should focus increasing attention on pre-diabetic populations.

Compared with the previously diagnosed diabetes participants, newly diagnosed diabetes participants had a higher level of blood sugar, blood fat and blood pressure, which is likely to be related to the lack of awareness and treatment. People with a clear diagnosis of diabetes tend to follow the doctor’s advice by taking regular medication, doing moderate exercise and following a low sugar diet^23^. People unaware of their diabetes follow their original life styles, which may not benefit their health.

### Influential factors

Similar to previous research^24^, this study demonstrates that the male gender, increasing age, higher BMI, and a family history of diabetes are all risk factors for diabetes. In addition, our study showed that central obesity (WC) is also a significant risk factor for both diabetes and pre-diabetes. It has been previously reported that an increase in WC leads to an increased risk of death^25^. Furthermore, people with a normal BMI but an excess WC, are more likely to have a metabolic disease^17^, and as an early warning signal of some chronic diseases, central obesity is more significant than BMI^26^. Previous research reported that higher education is a protective factor for diabetes, which may be related to the understanding of diabetes and the inclination to keep a healthy lifestyle^27,28^. In this study however, higher education appeared to be a risk factor for diabetes, which may be in fact associated with the earlier detection of the disease. In line with previous studies, we also showed that both not drinking and taking exercise frequently were both protective factors for diabetes^24^, and confirm that diabetes is associated with a high prevalence of hypertension and dyslipidemia^29,30^. Although the pre-diabetes group had a blood sugar level lower than that of a diabetic, the prevalence of hypertension, dyslipidemia and obesity were larger than in the normal group.

Metabolic diseases are serious chronic diseases with multiple complications, yet the awareness by participants of these diseases is still poor^12,31^, which was highlighted in our study where over 30% of adults with diabetes were still not aware of their hypertension status, and an even higher number were not aware of their dyslipidemia status. However, regardless of diabetes status, the poor control rates of hypertension and dyslipidemia may partly be attributed to noncompliance with drug therapy^32^. This could be based on a fear of side effects including worries about their effect on sexual performance, concerns about dependency, insufficient health insurance to cover costs, or a poor knowledge of the disease risks^13,31,33,34^.

In spite of increased vigilance in the detection of diabetes, the attention afforded to pre-diabetes is insufficient. To enhance the quality of general health and reduce the morbidity of diabetes, the Chinese government should actively encourage regular blood sugar monitoring.

### Study strengths and limitations

This study is the largest sample of diabetic and pre-diabetic patients in northeast China. The response rate was high, and the study was actually beneficial to the participants in that it included a medical exam. Limitations included the use of self-reported information, such as drinking, smoking and taking exercise, which may lead to recall bias. In addition, the study didn’t discriminate between diabetes type I and type 2, although most participants did have diabetes type 2. Moreover, all the participants were only recruited from the Jilin Province and therefore might not be comparable to other areas of China. Finally, blood lipids were measured from venous blood and not all participants had their blood lipids measured, notably those who clearly knew about their blood lipid status (such as those who had been diagnosed with dyslipidemia by doctors) and non-fasting subjects.

## Conclusion

The levels of pre-diabetes and diabetes in Northeast China that we show in this study indicate that the medical authority needs to increase their focus in this area. Given that the link between increases in blood sugar levels and the incidence of cardio-metabolic disorders, we highly recommended that participants with pre-diabetes and diabetes monitor their health regularly.

## Supplementary information

supplimetary information
