# Supplementary material for: The prevalence of pre-diabetes and diabetes and their associated factors in Northeast China: a cross-sectional study
Source: Sci Rep. 2019 Feb 21;9:2513. doi: 10.1038/s41598-019-39221-2 (PMC6385189; doi:10.1038/s41598-019-39221-2)
Supplement: Supplementary file 1 — supplimetary information [file 41598_2019_39221_MOESM1_ESM.pdf]

Supplementary information

Title: The prevalence of pre-diabetes and diabetes and their associated factors in Northeast China: a cross-sectional study.

RuiWang[a] Peng Zhang[b] Zhijun Li[d] Xin Lv[c] Hongfei Cai[a] Chunshi Gao[c] Yuanyuan Song[c]Yaqin Yu [c] **Bo Li[c*] Youbin Cui[a*]**

[a] Department of Thoracic Surgery, the First Hospital of Jilin University, Changchun 130021, China.

[b] Department of Neurology, Stroke Center, the First Hospital of Jilin University, Chang Chun130021, China.

[c] Department of Epidemiology and Biostatistics, Jilin University School of Public Health, 1163 Xinmin Street, Changchun, Jilin 130021, China.

[d]Department of Epidemiology and Biostatistics, Beihua University School of Public Health,

[*]Corresponding author:

Youbin Cui, Ph D

Department of Thoracic Surgery,

the First Hospital of Jilin University,

71 Xinmin Street, Changchun, Jilin 130021, China.

Tel:+86 81875310

E-mail: cuiyb@jlu.edu.cn

Bo Li, Ph D.

Department of Epidemiology and Biostatistics,

Jilin University School of Public Health,

1163 Xinmin Street, Changchun, Jilin 130021, China

Tel: ＋86 043185619451

E-mail: li_bo@jlu.edu.cn

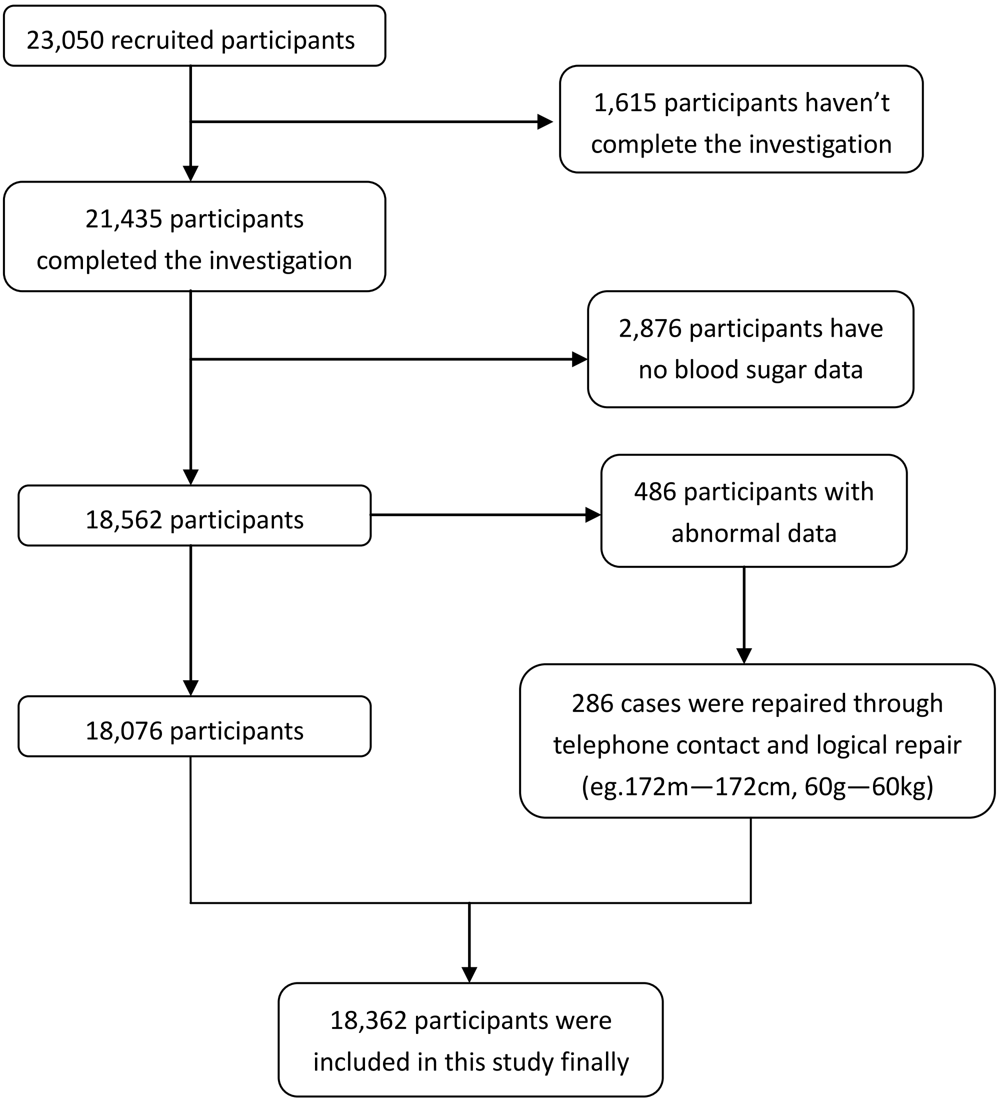

Figure 1: Sample processing flow chart

Personal health questionnaire

Code：□□/□/□□/□□□/□

（District / Street / Investigation point / Surveyed family / Investigator）

Name ： ____________________

Tel. number： ____________________

District / county: ____________________

Township / street / functional units: ____________________

Village / neighborhood committee / group： ____________________

Health Department of Jilin Province

College of public health, Jilin University

Jilin Center for Disease Control and Prevention

June 2012

Informed consent

The investigation of adult chronic diseases and its risk factors in Jilin province was led and coordinated by the Department of health of Jilin province. The school of public health of Jilin University and the Center for Disease Prevention and Control of Jilin Province has been jointly organized. The purpose is to grasp the distribution of the related risk factors of chronic diseases in different regions and different populations of the whole province. It provides a scientific basis for health departments to formulate and evaluate health policies and interventions. Your participation in this survey is the random selection we have chosen in the whole province. The survey includes questionnaire survey, anthropometry, blood pressure measurement, and electrocardiogram and blood biochemical examination. The blood biochemical examination required 3~5ml blood (empty stomach). All of the investigations and tests we have carried out are free of charge. If you agree to take part in the survey, we will give you the physical examination and test results as soon as possible after the completion of the investigation. Meanwhile, we guarantee that all the questions that may be related to your personal and family privacy in the investigation are strictly confidential. This survey will take about half an hour. Please forgive us for the inconvenience. If you have a problem that you don't understand, or if you have any suggestions, please bring it up and we will try our best to solve it.

If you agree to take part in our investigation, please sign it below. Thank you！

Signer's signature (cannot write by the index finger):

____

Date of filling:

____

Health Department of Jilin Province

College of public health, Jilin University

Jilin Center for Disease Control and Prevention

Start time:__________

Part 1: Demographics

A1 Date of birth: __________Year_____Month_____Day

A2 Sex: 1. male 2. female

A3 Nation: l. Han 2. Man 3. Mongolian 4. Korean 5.other__________

A4 Education level : 1. Primary school and below 2. Junior middle school 3. High school 4. undergraduate 5. graduate students and above

A5 Marital status: 1. married or cohabiting 2. single or separated 3. widowed or divorced 4. other__________

A6 Occupation: 1. students 2. manual workers 3. mental workers 4. retirees 5. Others

A7 Per capita monthly income of the family (Yuan): 1. <1000 2. 1000~ 3. 2000~ 4. 3000~ 5. 5000~ 6. unclear

Part 2: Smoking situation

Active smoking

B1 Do you smoke now? 1. yes 2. no

B2 You smoke average of______ cigarettes a day, a total of ______ took years

B3 If you have quit smoking, how many years have you been smoking cessation? ______ years

Passive smoking (Note: smokers jump to C1)

B4 Whether there is a passive smoking (Smoke inhaling smoke from other people for more than 15 minutes)? 1. almost no (jump to C1) 2. occasionally 3. often 4. everyday

B5 Passive smoking places (Multiple choices) 1. home 2. workplace 3. restaurants 4.entertainment places 5. other __________

Part 3: Drinking situation

C1 Do you drink now?？ 1. yes 2. no

C2 The frequency of drinking is： 1. <1 /week 2. 1~2 times/week 3. 3~5 times/week 4. 6~7 times/week

C3 Drinking type： 1. liquor 2. beer 3. wine 4. other__________

Part 4: Diet situation

D1 Whether the diet is regular？ 1. yes 2. no

D2 What is the taste of the diet？ 1. salty 2. light 3. moderate

D3 Eating habits: 1. meatarian diet 2. vegetarian diet 3. balanced diet 4. other

D4 What kind of staple food is the main type？ 1. rice 2. coarse cereals 3. flour 4. other__________

D5 How many staples are eaten on an average day? __________g

D6 Do you have breakfast every day？ 1. yes 2. no

D7 Food consumption frequency

D7a. Fresh Vegetables 1. eat regularly or almost every day 2. Sometimes 3. seldom or never eat

D7b. Fresh fruit 1. eat regularly or almost every day 2. Sometimes 3. seldom or never eat

D7c. Meat and poultry 1. eat regularly or almost every day 2. Sometimes 3. seldom or never eat

D7d. Aquatic product 1. eat regularly or almost every day 2. Sometimes 3. seldom or never eat

D7e. Eggs and bean products 1. eat regularly or almost every day 2. Sometimes 3. seldom or never eat

D7f. Milk and dairy products 1. eat regularly or almost every day 2. sometimes 3. seldom or never eat

Part 5: Physical activity

E1 What kind of transportation do you often use?？ 1. Bicycles, 2. Walking, 3. Bus or driving

E2 Does you often take exercise？ 1. Never 2. Sometimes 3. Often

E3 where do you take exercise？ 1. Home 2. Around the place of residence 3. to a special site (gym, etc.)

Part 6: Health condition

Number	System	Disease
**F1**	Infectious and parasitic diseases	1. hepatitis 2. Tuberculosis 3. other _____
**F2**	Tumor	1. liver cancer 2. lung cancer 3. gastric cancer 4. colon cancer 5. breast cancer 6. cervical cancer 7. prostate cancer 8. thyroid cancer 9. leukemia in 10. other ________
**F3**	Blood system and immune related diseases	1. anemia 2. rheumatism 3. other ________
**F4**	Endocrine, nutritional and metabolic diseases	1.diabetes 2.hyperlipidemia 3.hyperthyroidism 4. Osteoporosis 5. Gout 6. other ______
**F5**	Mental and behavioral disorders	1.schizophrenia 2.depressio 3. other________
**F6**	Nervous system disease	1. cognitive impairment 2. epilepsy 3. Parkinson's 4. other ______
**F7**	Ophthalmic and appendage disease	1. cataract 2. Glaucoma 3. other _______
**F8**	Circulatory disease	1. hypertension 2. cerebrovascular disease 3.angina pectoris 4. acute myocardial infarction 5. cor pulmonale 6. varicose veins of lower extremity 7.

		other _______
**F9**	Respiratory diseases	1. chronic obstructive pulmonary emphysema and 2. asthma 3. nasopharyngitis<br>4. chronic bronchitis 5. other _______
**F10**	Digestive system disease	1. gastric and duodenal ulcers, 2. fatty liver and 3. liver cirrhosis 4. cholecystitis, 5. gallstones 6. abdominal hernia 7. other __________
**F11**	The musculoskeletal system and connective tissue diseases	1. rheumatoid arthritis 2. intervertebral disc disease in 3. other ____
**F12**	Diseases of genitourinary system	1.nephritis 2.urolithiasis 3.prostatitis 4.gynecologic inflammation 5. breast disease 6. other ______
**F13**	Injury, poisoning, etc	1. injury (trauma, burn) 2. Poisoning 3. other ____
**F14**	Other	1. ____________ 2. __________

Hypertension control

F15 How long is the last time to measure the blood pressure distance? (Excluding the survey in this survey)

1. Within 30 days 2. Over 30 days 3. Never measured 4. Not clear

F16 Do you take measures to control your blood pressure? 1. yes 2. no

F17 What measures are taken to control blood pressure? (Multiple choices)

1. take medicine according to doctor's advice 2. control diet 3. moderate exercise

4. other, please specify __________

Blood glucose control

F18 How long is the last time to measure the blood sugar distance? (Excluding the survey in this survey)

1.Within 30 days 2. Over 30 days 3. Never measured 4. Not clear

F19 Do you take measures to control your blood sugar?

1. yes 2. no

F20 What measures are taken to control blood sugar? (Multiple choices)

1. take medicine according to doctor's advice 2. control diet 3. moderate exercise 4. other, please specify __________

Blood lipid control

F21 How long is the last time to measure the blood lipid distance? (Excluding the survey in this survey)

1.Within 30 days 2. Over 30 days 3. Never measured 4. Not clear

F22 Whether is your dyslipidemia diagnosed by a district or more hospital?

1. yes 2. No

F23 Do you take measures to control your blood lipid?

1. yes 2. no

F24 What measures are taken to control blood lipid? (Multiple choices)

1. take medicine according to doctor's advice 2. control diet 3. moderate exercise 4. other, please specify __________

Family history of major chronic diseases

F25 Did your grandparents, parents, and brothers and sisters have been diagnosed with the following chronic diseases by a county (District) or above hospital? (Multiple choices)

1. had not been diagnosed with any chronic disease
2. circulatory system diseases (hypertension, angina, myocardial infarction, cerebrovascular disease, etc.)
3. diabetes
4. respiratory system diseases (such as chronic bronchitis, emphysema, asthma)
5. tumor
6. hyperlipidemia
7. other, please specify _______________
8. unclear

Menopause (only for 35 years old and above)

F26 Do you have a menopause? 1. Yes, age of menopause:______ 2. no

The end time of the survey:__________

Part 7: Anthropometric results

G1 Height： __________ cm

G2 Weight： __________ kg

G3 Waist： __________ cm

G4 Hipline： __________ cm

G5 Somatic activity： 1. freely 2. Inability or involuntary tremor 3. paralysis 4. Limited activity

G6 Blood pressure： ________/________ （mmHg)

G7 Heart rate： __________times/min

G8 Electrocardiogram： 1. normal 2. Roughly normal 3. abnormal __________________________

G9 Blood biochemistry：
1. Blood sugar： __________mmol/L
2. TG： __________mmol/L
3. TC： __________mmol/L
4. LDL： __________mmol/L
5. HDL： __________mmol/L

Surveyor's signature： __________________________ **Date：** ______________________

Quality controller's signature： ____________________ **Date：** ______________________
